# Differences in Hospitalization Outcomes of Kidney Disease between Patients Who Received Care by Nephrologists and Non-Nephrologist Physicians: A Propensity-Score-Matched Study

**DOI:** 10.3390/jcm10225269

**Published:** 2021-11-12

**Authors:** Chien-Wun Wang, Yu Yang, Chun-Chieh Yeh, Yih-Giun Cherng, Ta-Liang Chen, Chien-Chang Liao

**Affiliations:** 1 Department of Anesthesiology, Shuang Ho Hospital, Taipei Medical University, New Taipei City 235, Taiwan; chien-wun@yahoo.com.tw (C.-W.W.); stainless@s.tmu.edu.tw (Y.-G.C.); 2 Department of Anesthesiology, School of Medicine, College of Medicine, Taipei Medical University, Taipei 110, Taiwan; tlc@tmu.edu.tw; 3 Division of Nephrology, Department of Internal Medicine, Changhua Christian Hospital, Changhua 500, Taiwan; 2219@cch.org.tw; 4 Department of Surgery, China Medical University Hospital, Taichung 404, Taiwan; b8202034@gmail.com; 5 Department of Surgery, University of Illinois, Chicago, IL 60637, USA; 6 Department of Anesthesiology, Wan Fang Hospital, Taipei Medical University, Taipei 116, Taiwan; 7 Anesthesiology and Health Policy Research Center, Taipei Medical University Hospital, Taipei 110, Taiwan; 8 Department of Anesthesiology, Taipei Medical University Hospital, Taipei 110, Taiwan; 9 Research Center of Big Data and Meta-Analysis, Wan Fang Hospital, Taipei Medical University, Taipei 116, Taiwan; 10 School of Chinese Medicine, College of Chinese Medicine, China Medical University, Taichung 404, Taiwan

**Keywords:** complications, hospitalization, kidney disease, mortality, nephrologists

## Abstract

The influence of physician specialty on the outcomes of kidney diseases (KDs) remains underexplored. We aimed to compare the complications and mortality of patients with admissions for KD who received care by nephrologists and non-nephrologist (NN) physicians. We used health insurance research data in Taiwan to conduct a propensity-score matched study that included 17,055 patients with admissions for KD who received care by nephrologists and 17,055 patients with admissions for KD who received care by NN physicians. Multivariable logistic regressions were conducted to calculate adjusted odds ratios (ORs) with 95% confidence intervals (CIs) for 30-day mortality and major complications associated with physician specialty. Compared with NN physicians, care by nephrologists was associated with a reduced risk of 30-day mortality (OR 0.29, 95% CI 0.25–0.35), pneumonia (OR 0.82, 95% CI 0.76–0.89), acute myocardial infarction (OR 0.68, 95% CI 0.54–0.87), and intensive care unit stay (OR 0.78, 95% CI 0.73–0.84). The association between nephrologist care and reduced admission adverse events was significant in every age category, for both sexes and various subgroups. Patients with admissions for KD who received care by nephrologists had fewer adverse events than those who received care by NN physicians. We suggest that regular nephrologist consultations or referrals may improve medical care and clinical outcomes in this vulnerable population.

## 1. Introduction

Kidney disease led to 1.2 million deaths in 2017 and is also one of the leading causes of years lived with disability [1,2]. Kidney diseases increase the risk of cardiovascular events, stroke, cognitive impairment, anemia, and electrolyte and bone disorders, accounting for substantial medical costs [3]. Subclinical chronic kidney diseases and acute kidney injury are commonly overlooked and regarded as harmless or reversible. However, there is accumulating evidence that changes in absolute serum creatinine are linked to greater mortality, development of chronic kidney disease, and increased healthcare expenditures [4,5]. Awareness, prevention, and early intervention are crucial to halt the progression of kidney diseases.

Physician specialty has an impact on patient outcomes and has been reported among cardiologists [6,7], gastroenterologists [8], neurologists [9], pulmonologists [10], and intensivists [11,12]. To date, only a few studies have examined the influence of physician specialty on the outcomes of kidney diseases [13,14,15]. Prior studies have demonstrated that early nephrologist consultation reduces the risk of 30-day mortality [14,16], lengths of intensive care unit and hospital stays [13], dialysis dependence at hospital discharge [14], and all-cause mortality at 6 months [14]. However, there are some study limitations in interpreting these findings, including relatively small sample sizes [13,15], restrictions to patients with acute kidney injury [13,14,15], and inadequate control for confounding factors [13,14]. In addition, whether nephrologists, as primary care providers, improve the outcomes of patients with kidney diseases remains unclear.

Accordingly, we used medical insurance claims data in Taiwan and conducted a population-based study to evaluate patients hospitalized for kidney diseases. Our purpose is to compare the in-hospital outcomes of kidney diseases between medical care from nephrologists and non-nephrologist (NN) physicians.

## 2. Methods

### 2.1. Source of Data

We used research data from the reimbursement claims of Taiwan’s National Health Insurance, which contain information on inpatient and outpatient medical services. Demographic characteristics, physicians’ primary and secondary diagnoses, treatment procedures, prescriptions, and medical expenditures were collected in the database. The insurance program covered more than 99% of people in Taiwan, and many scientific articles based on this database have been published in outstanding journals worldwide.

### 2.2. Ethical Approval

To protect patient privacy, the electronic database used in this study was decoded with patient identification scrambled for further academic access for research. Although the Ministry of Health and Welfare, Taiwan, exempts such uses from informed consent, the guidelines of the Declaration of Helsinki were obeyed during the execution of this study. This study was also approved by the Institutional Review Board of Taipei Medical University (TMU-JIRB-201509050).

### 2.3. Study Design

From the reimbursement claims of Taiwan’s National Health Insurance, we identified 67,246 patients with non-surgical admissions due to KD in 2008–2013, with 35,950 of them receiving inpatient care by nephrologists. For appropriate comparison and obtaining eligible study subjects, we used a propensity-score matching technique to select 17,055 patients receiving inpatient care by nephrologists and 6264 patients receiving inpatient care by NN physicians. Factors in the propensity-score matching included sociodemographic information (such as age, sex, and low income), types of KD, and history of disease (such as hypertension, diabetes, hyperlipidemia, mental disorders, ischemic heart disease, heart failure, liver cirrhosis, and chronic obstructive pulmonary disease). We compared the complications, mortality, intensity of care, length of hospital stay, and medical expenditures during admission due to KD between patients receiving inpatient care by nephrologists and those receiving inpatient care by NN physicians.

### 2.4. Measures and Definition

Based on previous studies, we defined the status of low income according to the regulations of the Ministry of Health and Welfare, Taiwan. In this study, the NN physicians included physicians with medical specialties in general medicine, family medicine, neurology, cardiology, gastroenterology, thoracic medicine, endocrinology, and infectious disease. The physician’s diagnosis and the International Classification of Diseases, Ninth Revision, Clinical Modification (ICD-9-CM) were used to identify coexisting medical conditions within the 24-month pre-admission period, including hypertension (codes 401–405), diabetes (code 250), hyperlipidemia, mental disorders (codes 290–319), ischemic heart disease (codes 410–414), heart failure (code 428), liver cirrhosis (codes 571.2, 571.5, and 571.6), and chronic obstructive pulmonary disease (codes 491, 492, and 496). Types of KD included acute glomerulonephritis, nephrotic syndrome, chronic glomerulonephritis, nephropathy, unspecified acute kidney failure, chronic kidney disease, renal failure, unspecified renal sclerosis, unspecified disorders from nephropathy, kidney infections, and hydronephrosis. During the index admission for KD, pneumonia (codes 480–486), septicemia (codes 038 and 998.5), urinary tract infection (code 599.0), mortality, length of hospital stay, and medical expenditures were considered study outcomes.

### 2.5. Statistical Analysis

To select appropriate study subjects for comparison, a propensity-score matched pair analysis was used to identify patients with admissions for KD who received care by NN physicians and nephrologists. A non-parsimonious multivariable logistic regression model was used to estimate a propensity score for patients receiving or not receiving care by nephrologists. In this model, the covariates included age, sex, low income, types of KD, hypertension, diabetes, hyperlipidemia, mental disorders, ischemic heart disease, heart failure, liver cirrhosis, and chronic obstructive pulmonary disease. We matched the patients who received care by nephrologists to the patients who received care by NN physicians using a greedy matching algorithm (without replacement), with a caliper width of 0.2 SDs of the log odds of the estimated propensity score. Categorical variables between patients who received cared by nephrologists and patients who received care by NN physicians were analyzed by using frequencies (percentages) and chi-square tests. The t-tests and means ± standard deviations were used to compare continuous variables between patients who received care by nephrologists and those who received care by NN physicians. We calculated the adjusted odds ratios (ORs) and 95% confidence intervals (CIs) of the outcomes of admissions for KD associated with physician specialty by using multiple logistic regressions. A subgroup analysis was also performed to examine the association between KD outcomes and physician specialty in the subgroups according to age, sex, number of medical conditions, and types of KD.

## 3. Results

Because propensity-score matching was used (Table 1), the standardized mean difference of every factor is <0.001, such as age, sex, and low income, hypertension, diabetes, hyperlipidemia, mental disorders, ischemic heart disease, heart failure, liver cirrhosis, and chronic obstructive pulmonary disease, and types of KD.

Compared with patients with KD who received care by NN physicians (Table 2), those with KD who received care by nephrologists had lower risks of in-hospital complications, including pneumonia (OR 0.82, 95% CI 0.76–0.89), acute myocardial infarction (OR 0.68, 95% CI 0.54–0.87), intensive care (OR 0.78, 95% CI 0.73–0.84), and 30-day mortality (OR 0.29, 95% CI 0.25–0.35). A shorter length of hospital stay (10.7 ± 12.2 days vs. 12.6 ± 32.2 days, *p* < 0.0001) and lower medical expenditure (1717 ± 2623 US dollars vs. 1929 ± 3525 US dollars, *p* < 0.0001) were found in patients who received care by nephrologists as compared to those who received care by NN physicians.

In the stratified analysis (Table 3), we found that care by nephrologists was associated with reduced adverse events during admissions for KD in the subgroups of females (OR 0.78, 95% CI 0.73–0.85); males (OR 0.70, 95% CI 0.65–0.76); and people aged 20–49 years (OR 0.70, 95% CI 0.53–0.93), 50–59 years (OR 0.70, 95% CI 0.58–0.86), 60–69 years (OR 0.73, 95% CI 0.64–0.83), 70–79 years (OR 0.72, 95% CI 0.66–0.79), and ≥80 years (OR 0.79, 95% CI 0.72–0.88). The association between care by nephrologists and reduced adverse events during admissions for KD was also significant in people with (OR 0.76, 95% CI 0.60–0.97) or without low income (OR 0.74, 95% CI 0.70–0.79), acute kidney failure (OR 0.61, 95% CI 0.56–0.67), and chronic kidney disease (OR 0.72, 95% CI 0.65–0.80). The adjusted ORs of adverse events during admissions for KD patients with 0, 1, 2 and 3 or more medical conditions were 0.83 (95% CI 0.71–0.96), 0.76 (95% CI 0.69–0.84), 0.71 (95% CI 0.64–0.79), and 0.72 (95% CI 0.64–0.80), respectively.

Table 4 showed the stratified analysis by specific medical conditions for the association between physician specialty and adverse events after the admission due to kidney diseases.

## 4. Discussion

In this study, we utilized a large patient sample and conducted meticulous analyses using propensity-score matching procedures to evaluate the association of care by nephrologists with the outcomes of kidney diseases. Our analyses showed that patients admitted for kidney diseases had better outcomes when receiving primary care from nephrologists, including lower risks of 30-day mortality, pneumonia, acute myocardial infarction, and intensive care, as well as shorter lengths of hospital stay and lower medical expenditures. These findings provide valuable implications for optimal care in patients with kidney diseases.

This study showed the better outcomes of patients admitted for kidney diseases with care by nephrologists, which is consistent with prior studies [13,14,15,16,17,18,19]. Of note, our study has several strengths compared to previous studies in evaluating the benefits of involvement of nephrologists in kidney diseases. First, our study included not only patients diagnosed with acute kidney injury [13,14,15,16,17,18,19] or admitted to the intensive care unit [13,15,16] but also those with chronic kidney diseases and glomerulonephritis, which increases the external validity of our results. In addition, the large patient sample of our cohort allows for the implementation of propensity-score matching to multiple covariates, which helps minimize potential confounding biases. Third, most prior studies focused on the effect of nephrologist consultations [13,14,15,16,18,19], which may be hardly generalizable to nephrologists as primary care providers. Fourth, the nationwide coverage of our dataset included patients receiving wide-ranging levels of hospital care, which was lacking in previous studies [14,15,16,17,18,19].

We proposed some possible explanations for the superior prognosis of medical care by nephrologists. First, nephrologists are more familiar with the courses of kidney diseases and are able to avoid preventable kidney injuries in the early stage of renal diseases, resulting in better renal functional reserve at discharge [17] and a lower overall mortality rate [14,15,18,19]. Second, nephrologists have better adherence to evidence-based practices to examine ideal candidates requiring renal replacement therapy and to initiate dialysis in a timely manner, which might better preserve renal function and reduce mortality due to uremia [16,20,21]. Third, nephrologists are knowledgeable about the latest guidelines for kidney disease therapies and have better adherence to evidence-based practices [20,21]. The vigilance of evidence-based practice has a positive effect on patient outcomes, including preserved renal function [20,21]. Fourth, patients with decreased renal function have impaired immunity and higher vulnerability to infections [22]. Nephrologists may change modifiable risk factors for nosocomial pneumonia, such as improving nutrition and controlling organ failure [23]. Fifth, chronic kidney disease is associated with an exceedingly high risk of coronary artery disease and causes a worse prognosis after myocardial infarction [24]. A study has shown that early and frequent care by nephrologists before the initiation of dialysis may reduce the post-dialysis occurrence of major cardiovascular outcomes through better control of anemia, fluid overload, and potassium homeostasis [25].

Our study showed that care by nephrologists was associated with a higher risk of urinary tract infection than care by general physicians. There are some possible explanations for this result. First, previous studies have demonstrated that specialists achieved a higher diagnostic accuracy for diseases within their specialty areas compared to that in general physicians and family physicians [26]. Nephrologists may have a higher level of awareness and familiarity regarding the manifestation and diagnosis of urinary tract infection, which results in a higher diagnostic rate [27]. Second, specialists are prone to pull cases toward their specialty [28], which may cause more frequent diagnoses of urinary tract infections while under the care of a nephrologist, namely, “ascertainment bias”. Third, patients referred to nephrologists may have more rapidly progressive and severe kidney diseases, which was associated with a higher incidence of urinary tract infection [29]. However, our research database lacked information about disease progression and severity.

The disparity in the in-hospital outcomes between nephrologists and general physicians may also result from patient factors. A large-scale survey showed that approximately 13.4% of patients did not comply with referrals to medical specialist care, which may reflect differences in demographic and socioeconomic attributes [30]. Lower socioeconomic status was linked to a higher risk of acute kidney injury and death from renal causes [31]. Second, among Taiwanese patients, there are obstacles to accessing the resources of renal supportive care due to the lack of structured and practical pathways, which may contribute to underrecognition and undertreatment of renal diseases [32]. Patients overcoming the barriers to specialist care have a higher level of support from family members and social networks [33], which may improve outcomes by ameliorating depressive affects, enhancing patient perception of quality of life, and improving patient compliance [34]. Third, patients seeking specialty services may have better health literacy and knowledge of common chronic diseases and receive timely renal care, which subsequently improved the outcome after admission [35].

The national prospective cohort study in the United States indicated that the incidence of hospitalizations for sepsis was 110/1000 patient-years in patients with renal dialysis [36]. Among 8937 patients with renal dialysis who received non-cardiac surgeries in Taiwan, 11% of them had postoperative septicemia [37]. In contrast, among 8803 patients who were admitted to the intensive care unit with sepsis, 730 (8.3%) patients had end-stage renal disease [38]. Septicemia was considered as one of common complications for patients with kidney disease, particularly in dialysis patients [39,40]. The results of our study provided similar phenomenon showing that about 12% of patients with renal hospitalization had septicemia.

Our stratified analyses showed that the reduced adverse events associated with care by nephrologists were consistent across various subgroups, except for patients with kidney infections. We considered that patients with more severe renal infections might be more likely to be referred to nephrologists, which could lead to a higher rate of in-hospital adverse events in the group who received care by nephrologists. In addition, kidney infection (acute pyelonephritis) represents the most severe form of urinary tract infection and is associated with a high risk of both morbidity and mortality [41]. This might override the benefit of nephrologist care in patient outcomes.

There are some limitations to this study. First, we have no information regarding lifestyle, laboratory measures (such as estimated glomerular filtration rate, serum creatinine and electrolytes), and medications for kidney diseases. Second, we did not have data with respect to hospital volume. General physicians and nephrologists in high-volume hospitals may have more resources to better diagnose and treat kidney diseases, which could improve patient outcomes [42]. Third, there were no data concerning physician experience, which may affect the quality of health care, patient behavior, and clinical outcomes [43]. Fourth, our analyses did not consider the severity of renal diseases or the level of renal functional reserve. This could cause selection bias, as patients with more severe kidney diseases may be transferred to medical centers or to care by nephrologists. Fifth, our dataset did not include people with subclinical renal diseases, because they may not seek standard medical care. In addition, residual confounding bias was always possible, although we adjusted for many potential confounders. Finally, we could not fully determine whether a patient had real kidney disease or myocardial infarction because there is no laboratory and detailed prescription items in this study, it is impossible to determine whether a patient had real kidney disease or myocardial infarction. The validity of diagnosis codes has been evaluated, such as chronic kidney disease [44], acute myocardial infarction [45], and pneumonia [46]. The positive predictive value of health claims data for chronic kidney disease defined as an estimated glomerular filtration rate <60 mL/min/1.73 m^2^ ranged from 60 to 89% [44]. Regarding acute myocardial infarction, the use of ICD-9-CM 410 yields a positive predictive value of 92% and a sensitivity of 88% based on the research database of Taiwan’s National Health Insurance [45].

In conclusion, patients admitted for kidney diseases had better in-hospital outcomes when they received medical care from nephrologists rather than NN physicians. Regular nephrologist consultations or referrals may improve medical care and clinical outcomes in this vulnerable population. This warrants future studies to better clarify the benefits of active and timely nephrologist care in clinical outcomes.

## Figures and Tables

**Table 1:** Characteristics of patients hospitalized due to kidney diseases received care by nephrologists and non-nephrologist physicians (after matching) *.

	NN Physicians (N = 17,055)		Nephrologists (N = 17,055)		SMD
Sex	n	(%)	n	(%)	
Female	10,114	(59.3)	10,114	(59.3)	<0.001
Male	6941	(40.7)	6941	(40.7)	<0.001
Age, years					
20–29	169	(1.0)	169	(1.0)	<0.001
30–39	275	(1.6)	275	(1.6)	<0.001
40–49	809	(4.7)	809	(4.7)	<0.001
50–59	1868	(11.0)	1868	(11.0)	<0.001
60–69	3725	(21.8)	3725	(21.8)	<0.001
70–79	5969	(35.0)	5969	(35.0)	<0.001
≥80	4240	(24.9)	4240	(24.9)	<0.001
Low income					
No	16,040	(94.1)	16,040	(94.1)	<0.001
Yes	1015	(5.9)	1015	(5.9)	<0.001
Types of kidney diseases					
Acute glomerulonephritis	90	(0.5)	90	(0.5)	<0.001
Nephrotic syndrome	201	(1.2)	201	(1.2)	<0.001
Chronic glomerulonephritis	453	(2.7)	453	(2.7)	<0.001
Nephropathy, unspecified	115	(0.7)	115	(0.7)	<0.001
Acute kidney failure	4249	(24.9)	4249	(24.9)	<0.001
Chronic kidney disease	3964	(23.2)	3964	(23.2)	<0.001
Renal failure, unspecified	476	(2.8)	476	(2.8)	<0.001
Renal sclerosis, unspecified	4	(0.02)	4	(0.02)	<0.001
Disorders from nephropathy	70	(0.4)	70	(0.4)	<0.001
Infections of kidney	7260	(42.6)	7260	(42.6)	<0.001
Hydronephrosis	173	(1.0)	173	(1.0)	<0.001
Medical conditions					
Hypertension	8938	(52.4)	8938	(52.4)	<0.001
Diabetes	6714	(39.4)	6714	(39.4)	<0.001
Hyperlipidemia	542	(3.2)	542	(3.2)	<0.001
Mental disorders	3936	(23.1)	3936	(23.1)	<0.001
Ischemic heart disease	3491	(20.5)	3491	(20.5)	<0.001
Heart failure	1981	(11.6)	1981	(11.6)	<0.001
Liver cirrhosis	359	(2.1)	359	(2.1)	<0.001
COPD	2897	(17.0)	2897	(17.0)	<0.001

COPD: chronic obstructive pulmonary disease; NN, non-nephrologist; SMD, standardized mean difference. * After matching by propensity score, the standardized mean difference between nephrologists and NN physicians in every factor is <0.001.

**Table 2:** The comparison of outcomes after admissions due to kidney diseases in patients received care by nephrologists and non-nephrologist physicians.

	NN Physicians (N = 17,055)		Nephrologists (N = 17,055)		Risk of Outcomes	
Outcomes after Admission	Events	%	Event	%	OR	(95% CI) †
30-day mortality	621	3.6	192	1.1	0.29	(0.25–0.35)
In-hospital complications						
Pneumonia	1633	9.6	1372	8.0	0.82	(0.76–0.89)
Septicemia	2105	12.3	2080	12.2	0.99	(0.92–1.05)
Pulmonary embolism	23	0.1	24	0.1	1.04	(0.59–1.85)
Urinary tract infection	3452	20.2	4058	23.8	1.24	(1.18–1.31)
Deep wound infection	33	0.2	29	0.2	0.88	(0.53–1.45)
Acute myocardial infarction	162	1.0	111	0.7	0.68	(0.54–0.87)
Intensive care unit stay	2011	11.8	1632	9.6	0.78	(0.73–0.84)
Medical expenditure, USD ‡	1929 ± 3525		1717 ± 2623		*p* < 0.0001	
Length of hospital stay, days ‡	12.6 ± 32.2		10.7 ± 12.2		*p* < 0.0001	

CI, confidence interval; NN, non-nephrologist; OR, odds ratio. † Adjusted for all covariates listed in Table 1. ‡ Mean ± SD.

**Table 3:** The stratified analysis for the adverse events after the admission due to kidney diseases associated with physician specialty.

		Adverse Events *				
		n	Events	Rate, %	OR	(95% CI) †
Female	NN physicians	10,114	1792	17.7	1.00	(reference)
	Nephrologists	10,114	1488	14.7	0.78	(0.73–0.85)
Male	NN physicians	6941	1838	26.5	1.00	(reference)
	Nephrologists	6941	1418	20.4	0.70	(0.65–0.76)
Age 20–49 years	NN physicians	1253	143	11.4	1.00	(reference)
	Nephrologists	1253	107	8.5	0.70	(0.53–0.93)
Age 50–59 years	NN physicians	1868	288	15.4	1.00	(reference)
	Nephrologists	1868	217	11.6	0.70	(0.58–0.86)
Age 60–69 years	NN physicians	3725	673	18.1	1.00	(reference)
	Nephrologists	3725	525	14.1	0.73	(0.64–0.83)
Age 70–79 years	NN physicians	5969	1344	22.5	1.00	(reference)
	Nephrologists	5969	1054	17.7	0.72	(0.66–0.79)
Age ≥80 years	NN physicians	4240	1182	27.9	1.00	(reference)
	Nephrologists	4240	1003	23.7	0.79	(0.72–0.88)
No low income	NN physicians	16,040	3437	21.4	1.00	(reference)
	Nephrologists	16,040	2749	17.1	0.74	(0.70–0.79)
Low income	NN physicians	1015	193	19.0	1.00	(reference)
	Nephrologists	1015	157	15.5	0.76	(0.60–0.97)
Acute kidney failure	NN physicians	4249	1627	38.3	1.00	(reference)
	Nephrologists	4249	1168	27.5	0.61	(0.56–0.67)
Chronic kidney disease	NN physicians	3964	1078	27.2	1.00	(reference)
	Nephrologists	3964	842	21.2	0.72	(0.65–0.80)
Infections of kidney	NN physicians	7260	665	9.2	1.00	(reference)
	Nephrologists	7260	651	9.0	0.98	(0.87–1.10
Others	NN physicians	1582	260	16.4	1.00	(reference)
	Nephrologists	1582	245	15.5	0.93	(0.77–1.13)
0 medical condition ‡	NN physicians	2620	503	19.2	1.00	(reference)
	Nephrologists	2620	438	16.7	0.83	(0.71–0.96)
1 medical condition ‡	NN physicians	5581	1112	19.9	1.00	(reference)
	Nephrologists	5581	907	16.3	0.76	(0.69–0.84)
2 medical conditions ‡	NN physicians	4900	1052	21.5	1.00	(reference)
	Nephrologists	4900	809	16.5	0.71	(0.64–0.79)
≥3 medical conditions ‡	NN physicians	3954	963	24.4	1.00	(reference)
	Nephrologists	3954	752	19.0	0.72	(0.64–0.80)

CI, confidence interval; NN, non-nephrologist; OR, odds ratio. * Adverse events included with 30-day mortality, pneumonia, acute myocardial infarction, intensive care unit stay. † Adjusted for all covariates listed in Table 1. ‡ Included hypertension diabetes, hyperlipidemia, mental disorders, ischemic heart disease, heart failure liver cirrhosis, and chronic obstructive pulmonary disease within the 24-month pre-admission period.

**Table 4:** The stratified analysis by specific medical conditions for the association between physician specialty and adverse events after the admission due to kidney diseases.

		Adverse Events *				
		n	Events	Rate, %	OR	(95% CI) †
No hypertension	NN physicians	8117	1739	21.4	1.00	(reference)
	Nephrologists	8117	1390	17.1	0.74	(0.68–0.80)
Hypertension	NN physicians	8938	1891	21.2	1.00	(reference)
	Nephrologists	8938	1516	17.0	0.75	(0.69–0.81)
No diabetes	NN physicians	10,341	2186	21.1	1.00	(reference)
	Nephrologists	10,341	1802	17.4	0.77	(0.72–0.83)
Diabetes	NN physicians	6714	1444	21.5	1.00	(reference)
	Nephrologists	6714	1104	16.4	0.70	(0.64–0.77)
No hyperlipidemia	NN physicians	16,513	3531	21.4	1.00	(reference)
	Nephrologists	16,513	2851	17.3	0.76	(0.72–0.81)
Hyperlipidemia	NN physicians	542	99	18.3	1.00	(reference)
	Nephrologists	542	55	10.2	0.50	(0.35–0.72)
No mental disorders	NN physicians	13,119	2762	21.1	1.00	(reference)
	Nephrologists	13,119	2230	17.0	0.75	(0.71–0.80)
Mental disorders	NN physicians	3936	868	22.1	1.00	(reference)
	Nephrologists	3936	676	17.2	0.71	(0.64–0.80)
No ischemic heart disease	NN physicians	13,564	2839	20.9	1.00	(reference)
	Nephrologists	13,564	2295	16.9	0.75	(0.71–0.80)
Ischemic heart disease	NN physicians	3491	791	22.7	1.00	(reference)
	Nephrologists	3491	611	17.5	0.71	(0.63–0.80)
No heart failure	NN physicians	15,074	3080	20.4	1.00	(reference)
	Nephrologists	15,074	2465	16.4	0.75	(0.70–0.79)
Heart failure	NN physicians	1981	550	27.8	1.00	(reference)
	Nephrologists	1981	441	22.3	0.74	(0.64–0.85)
No liver cirrhosis	NN physicians	16,696	3553	21.3	1.00	(reference)
	Nephrologists	16,696	2836	17.0	0.74	(0.70–0.79)
Liver cirrhosis	NN physicians	359	77	21.5	1.00	(reference)
	Nephrologists	359	70	19.5	0.88	(0.60–1.28)
No COPD	NN physicians	14,158	2822	19.9	1.00	(reference)
	Nephrologists	14,158	2279	16.1	0.76	(0.71–0.80)
COPD	NN physicians	2897	808	27.9	1.00	(reference)
	Nephrologists	2897	627	21.6	0.70	(0.62–0.79)

CI, confidence interval; COPD, chronic obstructive pulmonary disease; NN, non-nephrologist; OR, odds ratio. * Adverse events included with 30-day mortality, pneumonia, acute myocardial infarction, intensive care unit stay. † Adjusted for all covariates listed in Table 1.

## Data Availability

The data underlying this study is from the Health and Welfare Data Science Center. Interested researchers can obtain the data through formal application to the Health and Welfare Data Science Center, Department of Statistics, Ministry of Health and Welfare, Taiwan (http://dep.mohw.gov.tw/DOS/np-2497-113.html (accessed on 8 November 2021)). Under the regulations from the Health and Welfare Data Science Center, we have made the formal application (included application documents, study proposals, and ethics approval of the institutional review board) of the current insurance data. The authors of the present study had no special access privileges in accessing the data which other interested researchers would not have.
